# Country and community vs poverty and conflict: Teasing apart the key demographic and psychosocial resilience and risk factors for Indigenous clinic-referred children and adolescents

**DOI:** 10.1177/00048674231187315

**Published:** 2023-07-22

**Authors:** Alasdair Vance, Janet McGaw, Jo Winther, Selena White, Joseph P Gone, Sandra Eades

**Affiliations:** 1 Academic Child Psychiatry Unit and Developmental Neuropsychiatry Program, The Royal Children’s Hospital, The University of Melbourne, Parkville, VIC, Australia; 2 The Wadja Aboriginal Family Place, The Royal Children’s Hospital, Parkville, VIC, Australia; 3 Faculty of Architecture, Building and Planning, The University of Melbourne, Parkville, VIC, Australia; 4 Department of Anthropology, Harvard University, Cambridge, MA, USA; 5 Department of Global Health and Social Medicine, Harvard Medical School, Boston, MA, USA; 6 School of Population and Global Health, Faculty of Medicine, Dentistry and Health Sciences, The University of Melbourne, Parkville, VIC, Australia

**Keywords:** Australian Indigenous children and adolescents, psychosocial factors, social adversity status

## Abstract

**Objective:**

Indigenous young people are known to have adverse demographic and psychosocial factors affecting worse mental health outcomes and some household factors aiding resilience. In Australia, there has been no exploration of these factors in clinically referred Indigenous young people assessed in a culturally appropriate way.

**Methods:**

A total of 113 Indigenous children and adolescents, 217 non-Indigenous young people, age, gender, mental disorder symptom severity, symptom-linked distress and impairment matched, and 112 typically developing participants, age- and gender-matched were recruited. Cultural validity and reliability of the impairing symptoms in Indigenous young people were determined. Key demographic and psychosocial factors were compared across the three groups.

**Results:**

The Indigenous clinical group differed significantly from the other two groups that did not differ on three possibly protective measures examined. Key demographic and psychosocial risk factors in the Indigenous group differed significantly from the non-Indigenous clinical group which in turn differed from the typically developing participants. The three groups exhibited a progressively increased magnitude of difference.

**Conclusions:**

It remains imperative to nurture features that provide protection and enhance resilience for Indigenous young people and their communities. Indigenous status is linked to significant demographic and psychosocial disadvantage over and above that conferred by clinical impairment and its management. It is crucial that these features are managed and/or advocated for with those demographic and psychosocial factors of the greatest magnitude dealt with first. Future systematic investigations of the contribution of these key factors to mental health referral pathways, assessment and management are needed.

## Introduction

Mental illness is an independent risk factor for overall worse health status, premature death and its effects are known to be large, long-lasting and potentially trans-generational (Deferio et al., 2019). In children, approximately 47% of the variance in mental illness and 26% in their well-being are explained by demographic and psychosocial variables (Patalay and Fitzsimons, 2016). It is well known that demographic and psychosocial factors can affect the mental health and wellbeing of young people, either enhancing resilience or increasing risk (Australian Institute of Health and Welfare (AIHW), 2015; World Health Organization (WHO), 2014a). However, the relationship remains complex because demographic and psychosocial factors can confer both risk and resilience in different individuals (AIHW, 2015; WHO, 2014b).

Indigenous young people’s mental health is significantly worse: A much higher percentage report high or very high levels of psychological distress (33% vs 13%) and/or a long-term mental health condition (29% vs 16%), are hospitalized for injury or poisoning (37% vs 23%), or die from injury or poisoning (52% vs 18%), are hospitalized for intentional self-harm (5% vs 2%) or die from intentional self-harm (29% vs 7%) (AIHW data, 2018). Unsurprisingly, 53% of the health gap for Indigenous young people could be explained by five socioeconomic factors (employment and hours worked, level of schooling, work qualifications, housing adequacy and household income) and six health risk factors (smoking, binge drinking, fruit and vegetable consumption, body mass index and physical exercise) (AIHW, 2018). Importantly, a further 11% of the health gap could be explained the interaction between the socioeconomic and health factors (AIHW, 2018).

Given the devastating history of violent European settlement of the continent, the very persistence of Indigenous people in contemporary society is a remarkable testament to the resilience of their communities. At the same time, it is unsurprising that Indigenous people exhibit long-standing indicators of colonial distress. Indeed, there is a large extant literature examining many key demographic and psychosocial variables, including in Indigenous young people (see AIHW, 2020). A targeted selection relevant to this paper is now summarized (see Flaxman et al., 2009): Indigenous young people are more than twice as likely to live at home with a sole parent (overwhelmingly their mother) compared to non-Indigenous young people. They are more than 1.5 times as likely to have separated parents who are younger and poorer (Walter and Hewitt, 2012). They are more than 1.8 times as likely to have siblings less than 15 years of age living at home and more than twice as likely to have four or more siblings living at home (Australian Bureau of Statistics [ABS], 2016). They are more than twice as likely to have a parent educated to year 10 level or lower and a father who is unemployed. They are more than 1.3 times as likely to have a mother who is unemployed. They are more than twice as likely to have a household income of less than $500 per week. They are approximately 16 times more likely to be under the supervision of the youth justice system and 18 times more likely to be in detention, with Indigenous males four times as likely as females to be under supervision. Indigenous young people are more likely to be separated from their parents for longer periods of 1 month or more because of cultural kinship, multi-carer, child-rearing practices, overcrowding in the home being 3.6 times more likely and moving home more frequently being over three times more likely. They are more than 10 times more likely to be in out of home care with over 80% then living permanently away from their parents until the age of 18 years.

Three important underlying risk factors are known to influence the nuanced association between mental health, demographic and psychosocial factors: The over-representation of Indigenous young people in the lowest socioeconomic strata (Gale et al., 1990). The increasing ratio of adolescents to adults in the Indigenous population (Hunter, 1993; Offer and Schonert-Reichl, 1992). The ‘triple jeopardy’ of human rights denial for Indigenous peoples, human rights interdependence with health and separately with mental health for all people (Tarantola, 2007). All these multilayered factors help explain the adverse effects of worsening demographic and psychosocial factors on increased mental health conditions in Indigenous young people. However, in contrast, Zubrick et al. (2005) reported that high occupancy households of Indigenous young people are linked with better social and emotional well-being than those in low occupancy households. Similarly, Hewitt and Walter (2021) outlined that Indigenous young people’s general health and well-being, living with a lone parent and kinship networks, did not differ from those living in parenting couple households.

Accordingly, it is our objective to tease apart demographic and psychosocial risk and protective factors to enable more targeted, optimal and holistic care for Indigenous young people. Despite the importance of a biological, psychological and social formulation to compose a holistic mental health management plan, there has been no systematic exploration of demographic and psychosocial factors in clinically referred Indigenous young people compared to non-Indigenous young people and non-Indigenous typically developing young people. This is important to aid early recognition and identification of key demographic and psychosocial factors to enable targeted management planning. Future clinical research is also needed to investigate how these key factors affect referral pathways into health care, assessment, formulation, treatment planning and management outcomes. Accordingly, in this study, the Indigenous authors aim to carefully assess key demographic and psychosocial factors in clinic-referred Indigenous children and adolescents. The cultural validity and reliability of the impairing patterns of symptoms will be carefully and systematically determined by Indigenous mental health staff and Aboriginal Health Liaison Officers (AHLOs). A clinic-referred, age, gender, mental disorder symptom severity, symptom-linked distress and impairment-matched clinic-referred group of non-Indigenous young people will be used as a clinical control group. Age and gender-matched typically developing participant groups from the same 50 local primary and secondary schools as the non-Indigenous clinical group will be used as a healthy control group. The authors hypothesize that the known key Indigenous demographic and psychosocial variables will significantly differ in the Indigenous young people compared to the non-Indigenous and typically developing children and adolescents. Because groups are matched for clinical impairment, the authors hypothesize that some of these demographic and psychosocial variables may be affording resilience.

## Methods

### Participants

Totally, 113 Indigenous children and adolescents aged 6–16 years, were recruited from consecutive referrals to the Wadja Aboriginal Family Place and their tertiary hospital-based Indigenous mental health team over a 3-year period. The Indigenous young people, their families and community were cared for by the Victorian Aboriginal Health Service or Victorian Aboriginal Community Controlled Health Organisations and/or the Victorian Aboriginal Child Care Agency. They were referred for a range of difficulties that overlapped but can be grouped as follows: oppositional defiant rule-breaking behaviors (61%), impulse control problems (19%), depression and anxiety difficulties (15%) and other (namely learning problems (5%)). 67% of these children were medicated, with stimulant medication being the most common (81%).

An age, gender, mental disorder symptom severity, symptom-linked distress and impairment (Rutter et al., 1975) matched clinic-referred group of non-Indigenous young people (*N* = 217) aged 6–16 years, from 50 local primary and secondary schools recruited as a clinical control group over a 5-year period (see Table 1). These 217 children and adolescents were screened from a total sample of 574 children and adolescents identified by teachers and/or school support staff as having coping difficulties which then referred them to specialized university clinics in metropolitan Melbourne (Australia) over a 5-year period. All 574 young people were assessed but only 217 were age, gender, mental disorder symptom severity, symptom-linked distress and impairment matched to the Indigenous group. They were referred for a range of difficulties that overlapped but can be grouped as follows: oppositional defiant rule-breaking behaviors (60%), impulse control problems (21%), depression and anxiety difficulties (12%), and other (namely learning problems (7%)). 63% of these children were medicated, with stimulant medication being the most common (83%).

**Table 1.** Key demographic and clinical factors in the (1) Indigenous clinical (I), (2) non-Indigenous clinical (NI) and (3) typically developing participant (TDP) groups.

	1 I *N* = 113 Mean (SD)	2 NI *N* = 217 Mean (SD)	3 TDP *N* = 112 Mean (SD)	*F*	*p*	Group differences
Age	11.00 (3.12)	11.03 (3.44)	10.72 (2.67)	0.42	0.66	1 = 2 = 3
Gender M, F	87.26	154.63	73.39	2.93^a^	0.23	1 = 2 = 3^b^
**SAS**	**9.31 (1.95)**	**7.97 (1.86)**	**7.03 (1.62)**	**27.29**	**<** **0.0005**	**1** **>** **2** **>** **3**
Parent symp	1.66 (0.57)	1.60 (0.61)		0.86^c^	0.39	1 = 2
Parent distress	1.42 (0.69)	1.45 (0.66)		–0.34^c^	0.73	1 = 2
Parent impair	1.63 (0.55)	1.55 (0.59)		1.06^c^	0.29	1 = 2

SAS: social adversity status; Parent symp: parent symptom severity of their child; Parent distress: parent report of child stress due to symptoms; Parent impair: parent report of child impairment due to symptoms on Rutter and Graham Interview Schedule.

a 3 × 2 χ^2^.

b 2 × 2 χ^2^.

c Independent sample *t* test (df = 328).

Bold type indicates significant group differences.

The two groups did not differ with respect to their referring problems, their medication status or the community-based psychosocial interventions that had been trialed. All the children and adolescents met the inclusion criteria of living in a family home (and not in an institution) and attending normal primary and secondary schools. All had nonage-corrected Intelligence Quotients of 70 or above (Wechsler, 2003) and none had a neurological disease, endocrine disease, substance abuse/dependence disorders, personality disorders, bipolar, or psychotic disorders. There was no refusal to participate. The cultural validity and reliability of the impairing patterns of symptoms in the Indigenous group were carefully and systematically determined by Indigenous mental health staff *or* AHLOs, ensuring that each carer-identified pattern of symptoms and associated functional impairment was correctly interpreted. Trained interviewers (mental health staff) interviewed all the remaining caregivers of the non-Indigenous young people.

An age and gender-matched typically developing participant (TDP) control group (*N* = 112) were recruited from the same 50 local primary and secondary schools to determine if the social adversity status (SAS; Taylor et al., 1986) of the Indigenous and non-Indigenous clinical groups differ (see Table 1). This group manifested no *Diagnostic and Statistical Manual of Mental Disorders* (DSM) mental disorders.

### Measures

The Schedule for Affective Disorders and Schizophrenia for school-age children-present and lifetime version (K-SADS-PL) (Kaufman et al., 2000) is a structured diagnostic interview schedule based on *Diagnostic and Statistical Manual of Mental Disorders* (4th ed.; DSM-IV) criteria (0 = no information, 3 = threshold), with a parent version. It was used to determine DSM-IV mental disorders from a structured clinical interview with each child’s caregiver. It has proven clinical utility, reliability (inter-rater reliability kappa > 0.75 [kappa = 0.88 current sample]) and validity.

The Rutter and Graham Interview Schedule (Rutter et al., 1975) is a semi-structured clinical interview originally developed to ascertain mental disorders presence or absence from a parent’s perspective. It ascertains overall mental disorder symptom severity, symptom-linked distress, and impairment rated on a 0, absent; 1, mild; and 2, severe Likert-type scale. The Rutter and Graham Interview Schedule has good test–retest reliability (κ = 0.85), consistency (Cronbach’s α = 0.92) and concurrent validity.

The Parental Account of Childhood Symptoms (PACS) (Taylor et al., 1986) is a semi-structured clinical interview with a parent, carer or guardian which was originally developed as an instrument for the measurement of children’s behavior problems as experienced at home. A trained interviewer administered the demographic section of the interview. A SAS scale (range 3–) was formed from a total of family income level (1–2), mother’s educational level (1–2), single parent status (0–1), sibling size (0–) and broken home status (1–2). The PACS has been demonstrated to have adequate inter-rater reliability (κ = 0.69–0.96) and Cronbach’s coefficient alphas ranged from 0.87 to 0.89.^31^

The fourth edition of the Wechsler Intelligence Scale for Children (WISC-4) (Wechsler, 2003) was used. This provides verbal comprehension, perceptual reasoning, working memory, processing speed and full-scale scores of measured intelligence via 10 core subtests. The WISC-4 is well established with valid and reliable (Cronbach’s α > 0.80) normative data.

### Procedure

The clinical research protocol was Hospital Ethics Committee approved (2019.207/56941). All participants and their caregivers were given verbal and written information and written informed consent was obtained from each participant’s caregiver before entering the study. Testing occurred over one session (90 minutes maximum duration) with breaks as needed. Each parent and their child were assessed in separate rooms by different trained clinical researchers (a child and adolescent psychiatry Fellow; a probationary psychologist—both under approved supervision; an Indigenous child and adolescent psychiatrist; an Indigenous clinical psychologist; and AHLOs). During the session, the parent was interviewed using the PACS demographic section, Rutter and Graham Interview Schedule overall mental disorder symptom severity, symptom-linked distress and impairment scales and K-SADS-PL while his or her child completed the WISC-IV. The WISC-IV (10 core subtests) was administered by a probationary psychologist under the supervision of a registered psychologist. Paper versions of the psychometric measures were used.

### Statistical analysis

Data were analyzed using the Statistical Package for the Social Sciences (SPSS/SC). All dimensional variables analyzed were normally distributed. Missing data were filled in by contacting the participants in question. Participant demographic, psychosocial and key clinical characteristics were compared across the three groups using a one-way analysis of variance. Where the omnibus F was significant, the post hoc Studentized Newman–Keuls procedure was conducted (*p* < 0.05) to determine the source of this significance. Separate logistic regression analyses comparing each of the three groups to each other were conducted to determine variables that significantly predicted group membership. Chen et al.’s (2010) grading of odds ratio’s into small/medium/large magnitude of clinical effect was used. Independent sample *t*-tests were used to compare the Indigenous and non-Indigenous clinical groups on mental disorder symptom severity, symptom-linked distress, and impairment.

## Results

The Indigenous and non-Indigenous clinical groups had higher SAS than the typically developing group. Moreover, the Indigenous clinical group had a greater SAS than the non-Indigenous clinical group. DSM mental disorders and Intelligence Quotients for the three groups are reported in Vance et al. (2022).

The Indigenous clinical group demonstrated a small magnitude clinical difference from the typically developing participants and the non-Indigenous clinical group, which, in turn, manifested a small magnitude clinical difference from the typically developing participants for the following measures: less likely to be living with a biological parent(s), biological parent(s) less likely to be employed, more likely to be separated from biological parent(s) for greater than 1 month. The Indigenous clinical group demonstrated a medium-large magnitude clinical difference from the typically developing participants and a small magnitude clinical difference from the non-Indigenous clinical group, which, in turn, manifested a small-medium magnitude clinical difference from the typically developing participants for the following measures: biological parents more likely to be separated, the biological parent(s) more likely to have a lower level of education. The Indigenous clinical group demonstrated a large magnitude clinical difference from the typically developing participants and a small magnitude clinical difference from the non-Indigenous clinical group, which, in turn, manifested a large magnitude clinical difference from the typically developing participants for the following measures: biological parents having a lower level of income, biological parents involved in legal issues (see Tables 2 and 3).

**Table 2.** Key demographic psychosocial factors in the (1) Indigenous clinical (I), (2) non-Indigenous clinical (NI) and (3) typically developing participant (TDP) groups.

Key psychosocial factor	1 I *N* = 113 Mean (SD)	2 NI *N* = 217 Mean (SD)	3 TDP *N* = 112 Mean (SD)	Group differences
Cohabits (BBP, OBP, OSP, Fost)	22%,40%,17%,8% (13% kinship care)	50%,30%,12%,2% (6% grandparents)	85%,8%,6%,1%	**1** **<** **2** **<** 3***
Parent sep (1 = no, 2 = yes)	1.73 (0.45)	1.46 (0.50)	1.17 (0.38)	**1** **>** **2** **>** 3***
Sibship size (number in home)	2.99 (1.69)	2.52 (1.46)	2.51 (1.05)	**1** **>** **2** **=** 3*
Educ BM +/or BF (P, S, Tafe, T)	1%,58%,23%,18%	1%,53%,18%,28%	1%,43%,10%,46%	**1** **<** **2** **<** 3***
Emp BM +/or BF (E, P, UE, S)	27%45%17%,10% 1%	50%,35%,7%,4%, 3%	70%,26%,0%,0%, 4%	**1** **<** **2** **<** 3***
Income (<$30 K,30–50 K, > 50 K)	58%,15%,27%	35%,21%,44%	2%,20%,78%	**1** **<** **2** **<** 3***
Legal	28%	10%	1%	**1** **>** **2** **>** 3***
Sep from parents (No,< 1M,> 1M)	61%,6%,33%	75%,4%,21%	95%,5%,0%	**1** **>** **2** **>** 3***
OoH care	27%	7%	1%	**1** **>** **2** **=** 3***
Location (city, regional, remote)	49%, 41%, 10%	64%, 36%, 0%	68%, 32%, 0%	**1** **<** **2** **=** 3*
Number carers	2.85 (1.33)	1.56 (0.55)	1.34 (0.51)	**1** **>** **2** **=** 3*

BBP: both biological parents; OBP: one biological parent; OSP: one step parent and one biological parent; Fost: foster care; Parent sep: biological parents separated; Educ BM /or BF: education biological mother and/or biological father; P, S, Tafe, T: primary, secondary, TAFE, tertiary education; Emp BM +/or BF: employment biological mother and/or biological father; E, HD, P, UE, S: employed, home duties, pensioner, unemployed, student; Legal: involvement in legal system; Sep from parents: separation from parents, <1M, >1M: less than 1 month, greater than 1 month separation; OoH: out of home.

*** *p* < 0.0005; *p < 0.05.

Bold type indicates significant group differences.

**Table 3.** Key demographic psychosocial factors compared across the (1) Indigenous clinical (I), (2) non-Indigenous clinical (NI) and (3) typically developing participant (TDP) groups.

Factor	Group comparison	Wald	*p*	OR	95% CI
Cohabits	1:2	20.50	<0.0005	1.25	1.13–1.37
(BBP, OBP, OSP, Fost)	1:3	37.63	<0.0005	2.39	1.81–3.16
	2:3	24.16	<0.0005	1.77	1.43–2.18
Parent sep	1:2	17.48	<0.0005	3.21	1.86–5.55
	1:3	44.67	<0.0005	13.51	6.29–28.57
	2:3	26.56	<0.0005	4.18	2.43–7.19
Sibship size	1:2	5.05	0.03	1.12	1.01–1.23
(number in home)	1:3	4.91	0.03	1.30	1.03–1.64
Educ BM +/or BF	1:2	7.55	0.006	1.95	1.21–3.14
(P, S, Tafe, T)	1:3	21.19	<0.0005	5.00	2.52–9.90
	2:3	13.18	<0.0005	2.57	1.54–4.27
Emp BM+/or BF	1:2	7.54	0.006	1.23	1.00–1.43
(E, P, UE, S)	1:3	13.98	<0.0005	2.01	1.39–2.89
	2:3	9.44	0.002	1.53	1.17–2.01
Income	1:2	14.58	<0.0005	2.62	1.60–4.29
(<$30 K, 30–50 K, >50 K)	1:3	29.40	<0.0005	58.82	13.70–250.00
	2:3	19.21	<0.0005	23.26	5.68–90.91
Legal	1:2	18.78	<0.0005	3.09	1.86–5.15
	1:3	11.85	0.001	35.71	4.67–250.00
	2:3	5.46	0.02	10.53	1.46–76.92
Sep from parents	1:2	8.19	0.004	1.23	1.07–1.41
(No, <1M, >1M)	1:3	14.87	<0.0005	2.05	1.42–2.96
	2:3	9.55	0.002	1.77	1.32–2.55
OoH care	1:2	8.06	0.005	1.37	1.15–1.64
	1:3	12.15	<0.0005	14.93	2.31–100.00
Location	1:2	8.16	0.005	1.31	1.16–1.73
(city, region, remote)	1:3	8.89	0.005	1.39	1.21–1.88
Number carers	1:2	8.02	0.005	2.4	1.41–2.94
	1:3	8.54	0.005	2.5	1.49–2.98

BBP: both biological parents; OBP: one biological parent; OSP: one step parent and one biological parent; Fost: foster care; Parent sep: biological parents separated; Educ BM/or BF: education biological mother and/or biological father; P, S, Tafe, T: primary, secondary, TAFE, tertiary education; Emp BM +/or BF: employment biological mother and/or biological father; E, HD, P, UE, S: employed, home duties, pensioner, unemployed, student; Legal: involvement in legal system; Sep from parents: separation from parents; <1M, >1M: less than 1 month, greater than 1-month separation; OoH: out of home.

The Indigenous clinical group demonstrated a small magnitude clinical difference from the typically developing participants and a small magnitude clinical difference from the non-Indigenous clinical group, which, in turn, manifested no difference from the typically developing participants for the following measures: increased regional location for home address, increased number of siblings in the home and increased number of carers for the young people. The Indigenous clinical group demonstrated a large magnitude clinical difference from the typically developing participants and a small magnitude clinical difference from the non-Indigenous clinical group, which, in turn, manifested no difference from the TDPs for the following measure: increased out-of-home care placement for young people (see Tables 2 and 3).

## Discussion

The key demographic and psychosocial features of the Indigenous group are consistent with the extant epidemiological literature (ABS, 2016; AIHW, 2018, 2020; Anderson et al., 2018; Flaxman et al., 2009; Walter and Hewitt, 2012): higher SAS than the typically developing participant group, and also the non-Indigenous clinical group. However, these factors have not led to worse clinical impairment as the two clinical groups were matched for parent-reported mental disorder symptom severity, symptom-linked distress, and the degree of impairment due to the mental disorder symptoms. Their clinical homogeneity was further evident given they did not differ in the main types of clinical problems leading to their referral to mental health services, psychological management and/or the medications trialed at the time of their referral. The magnitude of the clinical effects between them (odds ratio [OR] range: 1.12–3.21) was small. In contrast, the magnitude of the clinical effects between the Indigenous and typically developing groups ranged from small to large (OR range: 1.30–58.82), as did those between the non-Indigenous clinical group and the typically developing group (OR range: 1.77–23.26). Three variables manifest the largest ORs between these groups with multiplicative effects compared to the two clinical groups: biological parents more likely to be separated, having a lower level of income and being involved in legal issues. Interestingly, the magnitude of the clinical effect was greater in the Indigenous compared to the non-Indigenous clinical group.

Two possible conclusions can be drawn from these results. The first is that Indigenous status is linked to significant demographic and psychosocial disadvantage over and above that conferred by clinical impairment and its management. This would confirm that upstream interventions to address the social determinants of health, which are integral to the *Close the Gaps* Strategy, are critical. But it also suggests that clinical care teams should be augmented by professionals who are best placed to intervene in addressing these discrepancies: AHLOs, Social and Emotional Well-being (SEWB) Officers, Social Workers and Occupational Therapists.

The second conclusion that could be drawn is that some psychosocial and/or demographic factors are not causing adversity but rather are affording resilience, as despite the significant overall differences in SAS there is no clinical difference between the Indigenous and non-Indigenous groups. A closer look at the findings reveals that the particular factors of biological parental separation, poverty and legal conflict have multiplicative effects and were significantly worse in the Indigenous group. But the increased regional location for home address, increased number of siblings in the home and increased number of carers for the young people seem to afford resilience.

It is important to recognize and explore those demographic and psychosocial features that may confer protection as resilience factors for Indigenous young people. SEWB is directly proportional to the network of relationships Indigenous young people have with their family, kinship network and community: Their connections to *Country*, Cultural practices, *Ancestral Spirits* and *Spirits* of *Country* are also crucial (Calma et al., 2017; Dudgeon et al., 2016; Hawthorne, 2018; Prehn and Ezzy, 2020). Increased number of carers for Indigenous young people may be a direct result of the extended family, kinship and community networks available to them (Dudgeon et al., 2016). And these carers frequently provide deep, secure attachments and add unique culturally-deep life skills for Indigenous young people (Prehn and Ezzy, 2020). This stands in direct contrast to a Western perspective that often emphasizes the attendant risks of multiple carers associated with insecure attachment and ad hoc internalization of life skills (Claessens and Chen, 2013). A similar argument can be made for increased numbers of siblings in the home. Similarly, a Western viewpoint often sees an increased regional location for the home address for Indigenous young people as linked to limited access to welfare, health, and educational services and employment opportunities. In contrast, such regional locations are often linked to Indigenous enclaves (‘villages’) where many culturally safe and appropriate resources are implicitly available for young people to learn from. Indeed Hopkins et al. (2014) and Gennetian et al. (2012) note that Indigenous young people that move from absolute and relative poverty into socioeconomically advantaged areas may experience substantial stresses linked to relative isolation from extended family, community, and cultural supports.

It is, therefore, imperative that Indigenous status is recognized early and identified as part of the clinical mental health assessment of young people and their families. Targeted holistic management can follow a comprehensive cultural and bio-psychosocial formulation that ensures demographic and psychosocial features are addressed (Bhat et al., 2020; Hunter, 2007; Twizeyemariya et al., 2017). This may involve social work, welfare/legal professionals and other health and mental health professionals working together in a culturally safe and appropriate way so all the factors outlined in a young person’s comprehensive formulation may be managed and/or advocated for. Clearly, those factors of the greatest magnitude should be tackled first. Likewise, those factors typically regarded as SAS ought to be recognized and supported by multidisciplinary teams as resilience factors instead.

From a research perspective, future systematic investigations of the contribution of key demographic and psychosocial factors of different magnitude to mental health referral pathways, presenting symptoms, diagnoses, psychosocial management strategies, and effective medications are needed. Then, the relative contribution of particular demographic and psychosocial features of a young person’s presentation can guide societal resource allocation to address those factors of greatest import. Brown et al. (2006) outlined the need to understand the complex interaction of social, biomedical and political processes affecting the etiology and morbidity of cardiovascular disease and for research methods to encompass these nuanced, multilayered factors in order to develop effective holistic management. This approach is sorely needed to aid Indigenous mental health.

The main limitation of this study is the limited definition of the demographic and psychosocial features examined. Although defined through clinical interviews in a culturally valid and reliable manner, there remain many further factors in this domain to be carefully and systematically investigated in future studies. Also, the possible complicating factor of Indigenous young people being cared for by non-Indigenous carers as opposed to carers from their kinship network is a limitation. This could be examined in detail in future studies. It is clear that the primary strength is our Indigenous young people being assessed by Indigenous mental health staff *or* AHLOs, ensuring that each impairing pattern of symptoms and associated functional impairment were correctly interpreted in the Indigenous group.

In conclusion, the findings of this study suggest that Indigenous status is linked to significant demographic and psychosocial disadvantage over and above that conferred by clinical impairment and its management. Upstream interventions to address the social determinants of health remain critical. But they also need to be part of downstream mental health care. It is crucial that the demographic and psychosocial adversity factors of the greatest magnitude are dealt with first and are managed and/or advocated for as part of treatment plans. It also remains imperative not to disrupt those features that provide protection and enhance resilience for Indigenous young people and their communities. Western healthcare has regarded regional location and multiple siblings and carers as indications of SAS, but these findings confirm recent work by Indigenous epidemiologists that argues they are resilience factors. Future systematic investigations of the contribution of these key factors to mental health referral pathways, assessment, and management are needed.
